# Development and validation of a highly dynamic and reusable picture-based scale: A new affective measurement tool

**DOI:** 10.3389/fpsyg.2022.1078691

**Published:** 2023-01-17

**Authors:** Ping Liu, Ya’nan Wang, Jiang’ning Hu, Lin’bo Qing, Ke Zhao

**Affiliations:** ^1^Business School, Sichuan University, Chengdu, China; ^2^College of Electronics and Information Engineering, Sichuan University, Chengdu, China

**Keywords:** affective ratings, authentic emotions, verbal scales, highly dynamic, multiple repeated measure, picture-based scale

## Abstract

Emotion measurement is crucial to conducting emotion research. Numerous studies have extensively employed textual scales for psychological and organizational behavior research. However, emotions are transient states of organisms with relatively short duration, some insurmountable limitations of textual scales have been reported, including low reliability for single measurement or susceptibility to learning effects for multiple repeated use. In the present article, we introduce the Highly Dynamic and Reusable Picture-based Scale (HDRPS), which was randomly generated based on 3,386 realistic, high-quality photographs that are divided into five categories (people, animals, plants, objects, and scenes). Affective ratings of the photographs were gathered from 14 experts and 209 professional judges. The HDRPS was validated using the Self-Assessment Manikin and the PANAS by 751 participants. With an accuracy of 89.73%, this new tool allows researchers to measure individual emotions continuously for their research. The non-commercial use of the HDRPS system can be freely accessible by request at http://syy.imagesoft.cc:8989/Pictures.7z. HDRPS is used for non-commercial academic research only. As some of the images are collected through the open network, it is difficult to trace the source, so please contact the author if there are any copyright issues.

## 1. Introduction

The concept of emotion is a complex neural and psychological phenomenon central to the organization of human social behavior (Balsamo et al., 2020). Emotions are critical to the individual; the broaden-and-build theory of positive emotions suggests that positive emotional experiences can broaden people’s momentary thought-action repertoires and help them build enduring personal resources. Negative emotions, on the other hand, narrow people’s attention, making them miss the forest for the trees (Fredrickson, 2001). Weiss and Cropanzano’s (1996) study found that mood affects memory, evaluative judgments, processing strategies, and social behaviors.

Emotions are short-term, unstable psychological states that arise in individuals (Cheng, 2021) and may vary substantially or even rapidly over a day (Mehrabian, 1996); their volatile and random nature has made it more difficult to research them. Scholars have mostly induced specific emotional states by means of appropriate and controlled stimulus materials (Marchewka et al., 2014), such as IAPS (International Affective Picture System); IADS (International Affective Digital Sounds); EMDB (Emotional Movie Database) to investigate the effects of emotions on various cognitive processes. Despite extensive research results have been obtained, some scholars still pointed out that evoked emotions are far from those felt by individuals in real life. Thus, those findings may not be generalizable (Zheng et al., 2012).

Emotion measurement is the basis for conducting emotion research. Emotions can be identified and measured by analyzing speech, facial expressions, self-report, mobile phone data, or physiological data (Lietz et al., 2019). The measurement of human affect can be approached from two fundamental angles: automatic identification and self-report methods (see Table 1 for details). Of these, automatic recognition methods try to infer affective information by measuring the user’s behaviors and physiological signals automatically (Broekens and Brinkman, 2013; Desmet et al., 2016; Sonderegger et al., 2016). The self-report methods require the user to provide affective information relying on certain instruments or means, including verbal, pictorial, animation-based, and questionnaire-based methods (Broekens and Brinkman, 2013).

**TABLE 1:** Comparison of different emotion recognition methods.

Method	Applicable	Cost	Duration	Result	RM
PI	Laboratory	High	3–5 min	Objective	EMG
EB	Laboratory/Public space	High	Real-time	Objective	Facial recognition
VB	Various occasions	Low	3–5 min	Subjective	PANAS

PI, means physiological indicator measures; EB, means external behavioral measures; VS, means verbal scales; RM, means represent method; EMG, means electromyography.

While the above methods have played an important role in emotion research, they have some limitations. Take the automatic identification method as an example, subjects must wear extensive electrophysiological equipment during physiological indicator measurements, which may have problems such as complicated operation processes, high implementation costs, and difficulties in analyses. Subjects are measured in real-time in the case of external behavioral measures, which may violate personal privacy and cause resistance. In addition, the above methods are mainly used in experimental scenarios rather than daily emotion measurements.

Self-reporting is the most widely applied method to gather subjective information about an emotional experience (Reisenzein, 1994; Betella and Verschure, 2016; Sonderegger et al., 2016). Verbal scales usually use a variety of emotion adjectives to measure subjects’ emotional states (e.g., PANAS uses 20 items to measure subjects’ positive and negative emotions); in this way, we can obtain a wealth of information. However, it is noteworthy that this method is mainly suitable for a single test owing to a large number of items and the duration of the test. Emotions are transient and short-lasting phenomena (Gross, 1998), the day-by-day emotional experience of the subject cannot be captured by this single assessment (Pollak et al., 2011).

Employees at all levels are exposed to a variety of emotionally challenging events (Alam and Singh, 2019); continuous attention to individual emotional status and performing high-frequency emotion measurement has become a hot issue for companies and scholars. As existing emotion measures are not suitable for continuous measurement, there is an urgent need to develop simple, efficient, and applicable tools to assess emotions.

A picture is worth a thousand words (Wichmann et al., 2002). The pictorial scale is an instrument that makes use of image-based elements to convey the meaning of its items (Sauer et al., 2021), which are short (Kunin, 1998); intuitive (Baumgartner et al., 2018); repeatable; low linguistic dependence and uncomplicated to measure (Sauer et al., 2021). From this, we can be reasonably sure that the pictorial scales may serve as a new tool for continuous measurement.

Given that most studies have used pictures as emotional stimuli, and even though some scholars have developed picture-based scales for specific purposes, the underlying mechanism has not been discussed. This paper first compiled domestic and international studies on the relationship between emotions and pictures and found that emotion mediation theory, psychological projection techniques, and picture-based scales can provide some support for the assumption that pictures express emotions.

### 1.1. Emotional mediation theory

Music-color synaesthesia is a fascinating neurological phenomenon. Scholars have discovered that although only a small proportion of people have such synaesthesia, recent evidence suggests that self-reported non-synaesthesia exhibit robust and systematic music-to-color associations (Whiteford et al., 2018). Palmer et al. (2013) reviewed the Direct Connection Hypothesis and Emotion Mediation Hypothesis and explained the music-color relationship. Through three experiments, they discovered that music-to-color associations are mediated by common emotional associations. According to the Emotion Mediation Hypothesis, people have emotional associations with stimuli that constitute one of the fundamental bases on which cross-modal associations are established (Spence, 2019; Liang et al., 2021). People associate color with music in ways congruent with the emotions they spontaneously perceive in the music, or with emotions connected with memories or imagery within themselves while listening (Lindborg and Friberg, 2015). Analysis of emotional mediation mechanisms reveals that there is some degree of correspondence between emotion and color. Albertazzi et al. (2015) explored the existence of cross-modal associations in the general population between a series of paintings and a series of clips of classical (guitar) music, and discovered the existence of cross-modal associations between highly complex stimuli. Reviewing the related studies of emotion mediation theory, we concluded that there is a certain correspondence between emotion and color or painting; based on this logic, there may also be a certain correspondence between emotion and pictures.

### 1.2. Psychological projection technique

The psychological projection technique is regarded as one of the three major psychological testing techniques. It assumes that most of the structures of personality are in the subconscious mind, and one can often reveal their desires, needs, and motivations hidden in the subconscious mind when faced with an ambiguous stimulus situation (Peng, 2006). So with the help of this technique, subjects’ performance, perceptions, emotions, personality traits, etc., can be assessed indirectly. For example, in the Rorschach inkblot test, the researcher will present a standard inkblot diagram to the subject; by recording what the subject associates with, researchers can analyze the subject’s personality traits and affective.

The functioning mechanism of the psychological projection technique shows that subjects will project their own affect onto the selected images, thus, we can infer the current affective state of the subjects through those images. Compared with the self-report method, this technique has a certain degree of concealment; thus, when implemented, it encounters relatively less resistance in gathering accurate information (Zhou, 2006). Nevertheless, few scholars have used this technique to identify subjects’ affect due to the lack of objective criteria and the time-consuming and demanding nature of the test (Fang et al., 2010).

### 1.3. Picture-based scales

Much of the content of emotional experience is inherently per-verbal (Zhou, 2006). Kensinger and Schacter (2006) revealed that verbal stimuli lead to left-lateralized activation, whereas pictorial stimuli lead to right-lateralized or bilateral activation; thus, pictorial stimuli may lead to automatic processing vs. elaborative, associative, or conceptual processing compared to verbal stimuli. Images may induce an instinctive response (Lang, 1995); thus, for the presentation of individual emotional states, the use of pictures, drawings, and metaphors is more effective than verbal definition and description (Diem-Wille, 2001).

The first studies using pictures instead of words can be traced back to the Self-Assessment Manikin (SAM) devised by Lang et al. (1988). The SAM scale is a non-verbal picture measurement technique with three dimensions of valence, arousal, and dominance, including five cartoon portraits for each dimension. When measuring, subjects can either choose any figure or an intermediate state between the two figures, which results in a 9-point scale, whereby 1 represents the lowest level of the dimension and 9 represents the highest level of the dimension. Since the SAM scale overcomes issues with verbal scales, such as the need for time, effort and the ability to interpret written language (Obaid et al., 2015), and has a validity comparable to that of the verbal scale (Bradley and Lang, 1994; Kolakowska et al., 2020), researchers have primarily used it to assess changes in affect caused by a particular event rapidly. Despite the widespread use of the SAM scale in emotion research, the scale has been criticized since its design for being too sketchy, oversimplified, and unaesthetic (Sonderegger et al., 2016). Some researchers have also indicated that there is still an unresolved issue that the three dimensions need to be explained in detail before it was used (Broekens and Brinkman, 2013; Obaid et al., 2015; Wong et al., 2021).

Since the SAM scale can be outdated, subsequent scholars started to develop new pictural scales for emotion measurement (Betella and Verschure, 2016; see Table 2 for details).

**TABLE 2:** Picture-based scales for emotional measures.

Scales	References	Display format	Type of emotion	Items
Russkman IM	Sánchez et al., 2006	Humanlike-face	2 dimensions	1
AffectButton	Broekens and Brinkman, 2013	Humanlike-face	3 dimensions	1
EmoCards	Desmet et al., 2001	Humanlike-face	2 dimensions	1
Smileyometer	Read, 2008	Humanlike-face	1 dimensions	1
Affective slider	Betella and Verschure, 2016	Humanlike-face	2 dimensions	2
LFS (or SLFS)	Obaid et al., 2015	Humanlike-face	Discrete emotions	1
LE-PANAVA	Schreiber and Jenny, 2020	Humanlike-face	2 dimensions	10
Charaterized face series	Kunin, 1998	Humanlike-face	1 dimensions	1
SAM	Lang, 1980	Humanlike-face and body	3 dimensions	3
Pick-A-Mood	Desmet et al., 2016	Humanlike-face and body	2 dimensions	1
LEM	Huisman, 2010	Humanlike-face and body	Discrete emotions	1
PrEmo1	Desmet et al., 2001	Humanlike-face and body	Discrete emotions	1
PrEmo2	Desmet, 2002	Humanlike-face and body	Discrete emotions	1
Sorémo	Girard and Johnson, 2009	Humanlike-face and body	Discrete emotions	1
AniSAM	Sonderegger et al., 2016	Humanlike-face and body	2 dimensions	1
AniAvatar	Sonderegger et al., 2016	Humanlike-face and body	2 dimensions	1
MAAC	Manassis et al., 2009	Humanlike-face and body	Discrete emotions	16
AffectGrid	Russell, 1989	Grid	2 dimensions	1
Feeltrace	Covie et al., 2000	Grid	2 dimensions	1
PAM	Pollak et al., 2011	Photographs	2 dimensions	1
Ottawa mood scales	Wong et al., 2021	Humanlike-face and cartoon scenes	Discrete emotions	5

In the Display format column, Huamlike-face refers to the picture with facial expressions alone, Huamlike-face and body refers to the picture that includes both facial expressions and body movements. Humanlike-face and cartoon scenes refer to the picture that combines facial expressions and cartoon scenes. In the type of emotion column, one dimension is valence, two dimensions are valence and arousal, and three are valence, arousal, and dominance. Discrete emotions are specific emotions such as anger, happiness, sadness, etc.

**FIGURE 1:**
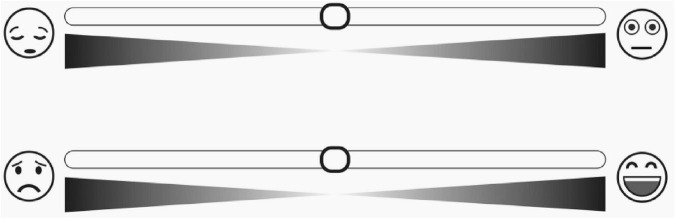
The affective slider.

A review of existing studies found that picture-based scales mainly express particular emotions with facial expressions and body movements, which have the problems of direct and single measurement form and lack of rigorous caricature design. For example, the Affective Slider (AS) is a new digital self-assessment scale composed of two slider controls that measure basic emotion in terms of pleasure and arousal (Betella and Verschure, 2016; see Figure 1). Although the authors claim that AS does not require written instructions and can replace SAM in the self-reporting of pleasure and arousal, the rationale for selecting expressions is not described during the tool development, which may affect the instrument’s reliability.

In addition to measuring emotions with the help of cartoons and expressions, there are also tools that use real pictures for emotion testing, such as PAM (Photographic affect meter). The PAM is a picture-based emotion measurement instrument in which subjects are asked to select 1 image from 16 images with different themes and contents that best match their current emotions (Pollak et al., 2011).

The design of PAM provides essential ideas for our study. Yet, it is worth noting that there are still some shortcomings of PAM. First of all, PAM does not design a strict picture scoring process, merely marking pictures based on the subject’s choice of pictures and reported emotions; thus, the reliability of the labels is questionable. Secondly, the test interface presents multiple types of images, such as people, animals, and scenes simultaneously, through which uncontrollable factors such as personal preferences may affect the accuracy of the test results. Last but not least, PAM contains just 100 images, which is small compared to a single presentation of 16 images, and may face a higher duplication ratio.

### 1.4. The present research

The repeated measurement of moods and emotions with high frequency is common in ambulatory psychological and psychophysiological assessment (Betella and Verschure, 2016). Related studies have widely used self-report methods for repeated measures and have made some research progress. However, we face the dilemma of insufficient tools because few standard psychological inventories can be transported wholesale into the daily diary format (Cranford et al., 2006).

In the present research, we investigate individuals’ authentic emotions and develop a highly dynamic and reusable picture-based scale that measures changes in affective state. Compared with the existing emotion measure tools, HDRPS has a novel format, diverse content, and an extensive capacity material library, allowing for efficient measurement of individual emotions.

**FIGURE 2:**
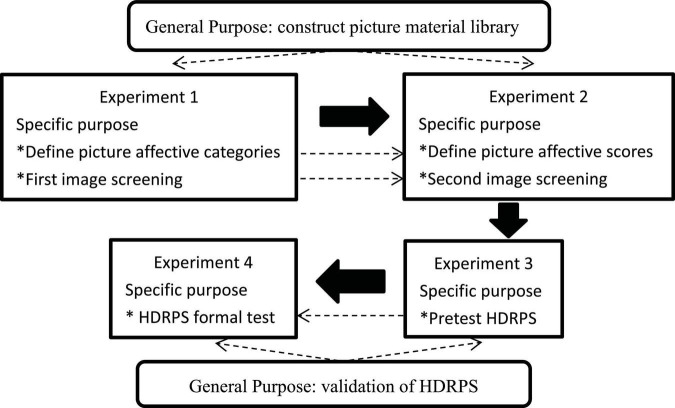
Experimental procedure and purpose. The dotted lines represent the intrinsic link between the experiment objectives, and the solid arrows indicate the study order.

In this research, four experiments were conducted in order to construct a highly dynamic picture-based scale (the details can be seen in Figure 2), of which Experiments 1 and 2 were aimed at constructing a picture material library. Based on dimension theories of emotion, we classified emotions into two dimensions: valence and arousal. Referring to Mehrabian’s (1996) related study for conceptual definition, valence refers to negative vs. positive affective states, which generally vary from cruelty, humiliation, disinterest, and boredom to excitement, relaxation, love, and tranquility. Arousal refers to the level of mental alertness and physical activity, and generally changes from sleep, inactivity, boredom, and relaxation to high arousal states such as wakefulness, bodily tension, strenuous exercise, and concentration. Experiments 3 and 4 were designed to validate picture-based scales applied to reality contexts. Considering that organizational science frequently conceptualizes emotion in terms of positive affective and negative affective (Weiss and Cropanzano, 1996), referring to the related study by Watson et al. (1988), we developed picture-based scales with valence rating as a cue. Then, to verify the validity of the picture-based scale, the semantic scale PANAS and the picture scale SAM were selected as the calibration scales. Further, to ensure the successful conduct of the study, we developed our own Picture Evaluation System (hereinafter referred to as PES)^1^ and Picture Based Scale Validity Verification System (hereinafter referred to as PSVS)^2^ to assist in completing the experiments.

## 2. Experiment 1: Expert evaluation of image material

### 2.1. Methods

#### 2.1.1. Materials

Referring to the internal architecture of NAPS (Nencki Affective Picture System) and OASIS (Open Affective Standardized Image Set), we established the framework of the material library containing people, animals, plants, objects, and scenes.

To facilitate image retrieval, we referred to a related study by Marchewka et al. (2014) for artificial image classification with the following criteria. Pictures in the “people” category were described as those containing visible, living human bodies or isolated parts of the human body. Pictures in the “animals” category were described as containing visible animals (images in this category could contain human body parts in the background). The “plants” category was described as plants in the growth cycle without humans or animals present. The “objects” category was described as natural or artificial objects with no human or animal present, in a non-growth cycle. Finally, the “scenes” category was described as images depicting a wide range of natural and man-made scenery, panoramas, or terrain without humans or animals visible.

Considering that picture screening would be continuously conducted during the construction of the material library, 42 college students were recruited to collect a wide range of color images on the Open Network. In order to expand the search scope as much as possible and reduce the repetition rate of images, we compiled our own search terms and asked participants to search for pictures based on the given terms. Since different states of the same object may represent different emotional states (e.g., calm sea, rough sea), each item was required to have no less than 10 images that cover different levels of valence and arousal. According to the search requirements, a total of 22,054 original images were eventually collected.

Given that the original images have different sizes and formats, we used OpenCV and Visual Studio 2015 to standardize the images into JPG format images with 512*512-pixel size. In view of the fact that physical properties may impact emotional judgments (e.g., Lakens et al., 2013; Carretié et al., 2019), we remapped the channel gray values of “objects” and “scenes” with the help of RGB curves, and tuned seven pictures with large color differences based on the original image.

Two Ph.D students and two master students from the research team evaluated the usability of the standardized processed pictures, while referring to relevant studies by Out et al. (2020) to develop usability evaluation rules. First, image content is complete and clear, without watermarks or text logos. Second, the emotional meaning is straightforward and does not require complex cognitive processing. Last but not least, pictures do not contain cultural sensitivities, and have no obvious stimulus elements that can quickly elicit an effective response from individuals. A total of 1,289 sets of images, 8,933 pictures, were remaining at the end of the usability evaluation.

#### 2.1.2. Participants

To ensure the scientific validity of the image labels, strict criteria for subject recruitment were established. First, subjects were required to have a Ph.D or higher study experience and relevant research experience in affective science. For the sake of convenience, 14 doctoral students from Sichuan University and Yunnan University were invited to participate in the experiment, including 7 males and 7 females, with an average age of 27.77 years. Secondly, the subjects were required to have normal color vision and psychiatric status. The Ishihara Color Blindness Test, Self-Rating Anxiety Scale (SAS; Zung, 1971a), and Self-Rating Depression Scale (SDS; Zung, 1971b) were performed in turn. The results showed that all of the subjects had normal color vision, the SAS scores below 50 and the SDS scores below 53, which were at the normal level of Chinese (Li et al., 2020), thus, all of the 14 subjects were qualified as experts.

The experiment process and evaluation indexes were explained in detail before the experiment started. The experts were asked to log in to the PES, sign an informed consent form, and receive certain compensation after the experiment was completed.

#### 2.1.3. Procedures

To reduce the influence of mental fatigue on the rating results, 14 experts were randomly and equally divided into two groups, A and B. The 1,289 sets of images were randomly divided into tasks 1 and 2 (task 1 contained 645 sets of images and task 2 contained 644 sets of images). Experts in groups A and B completed the rating tasks of task 1 and task 2, respectively. The experiment was conducted for 5 consecutive days, and the daily evaluation duration was about 3–4 h.

Before the experiment started, the expert logged into the PES, which automatically played an operation video and explained the experiment process and rating index in detail (the expert can choose to skip the session in the second time). The formal experiment consisted of three sessions. The first was affective self-reporting; in order to prevent personal emotions from influencing the assessment results, the PANAS scale and SAM scale (valence and arousal) were used to measure the affective state of the expert before the experiment began, and the test results were used as the rationale for the validity or otherwise of the rating data. The second was the pre-test practice, in order to ensure that the expert adequately understood the rating indexes and the operation process, a total of 5 sets (5 images/set) of pre-test practice were designed (the expert can choose to skip this section in the second time). The third was picture evaluation, considering that judging several different kinds of pictures at once can bias the obtained for each single category, and that scoring is stable and reliable when comparing a given category (Dan-Glauser and Scherer, 2011); the pictures were presented in sets, one by one, during the experimental process. Since the rapid conversion of rating indexes tend to influence the stability of results, every 15 sets of pictures were randomly packaged into one unit. During the formal assessment, experts first evaluated the valence of all pictures in the unit, and then all pictures were presented again to rate their arousal in order. Referring to OASIS, this study did not set a fixed time for the rating of the pictures but only asked the experts to answer as soon as possible, based on their intuition, without excessive thinking. At the same time, to eliminate the influence of mental fatigue on the rating results, an obligatory rest period was set, requiring experts to take at least a 3-min break after completing each of the 3 unit rating tasks.

### 2.2. Statistics

Reliability measures, also known as reliability tests, are usually measured by calculating the Cronbach coefficient of the scale (Baumgartner et al., 2019), which is generally within the range of 0.65–0.80 with a minimum acceptable level above 0.50 (Cook et al., 2018). Here we used Cronbach’s alpha coefficient to measure the reliability of the experts’ ratings.

Besides, introducing the concept of identity ratio. The identity ratio is the percentage of all participants who believe that the image belongs to this emotion type compared to the total number of evaluators (Wang and Chu, 2013). We referred to some scholars’ criteria (Li, 2014) and used a 60% identity ratio as the screening rule to further improve the quality of the materials.

### 2.3. Results

#### 2.3.1. Reliability measures

After excluding the rating data given when the experts’ affects were abnormal, the Cronbach’s alpha of the valence and arousal ratings of the experts in groups A and B were calculated separately. Table 3 shows that the Cronbach’s alphas of all variables were higher than 0.8, except for the arousal of group A, which was lower than 0.7. Based on this, we concluded that there is some internal consistency in the expert evaluation results.

**TABLE 3:** Consistency of expert ratings.

Group	Cronbach’s alpha of valence	Cronbach’s alpha of arousal
Group A	0.833	0.618
Group B	0.925	0.863

#### 2.3.2. Identity ratio of ratings

Following the way Pilar et al. (2012) classified valence and arousal, images with valence ratings of 1–3, 4–6, and 7–9 were labeled as negative, neutral, and positive in order, and images with arousal ratings of 1–3, 4–6, and 7–9 were labeled as low, medium, and high in order. The identity ratio of each image was calculated separately based on the affective labels of the images (valence, arousal). Pictures were screened according to 60% criteria (Li, 2014), and finally, a total of 1,269 sets and 5,046 pictures passed the evaluation.^2^

Considering the relatively small number of participants in the evaluation, more subjects were recruited for the affective judgments in experiment 2 in order to improve the stability of the affective labels and the reliability of the affective ratings.

## 3. Experiment 2: Of professional evaluation of image material

### 3.1. Methods

#### 3.1.1. Materials

The professional evaluation materials were 5,046 pictures that passed the Experiment 1, including 549 images of people, 974 images of animals, 816 images of plants, 998 images of objects, and 1,709 images of scenes.

#### 3.1.2. Participants

Considering that the purpose of this experiment is to refine the picture labels and further improve the scientificity of the picture scores, just as in Experiment 1, we limited the subject’s majors and health conditions. College students majoring in human resource management, psychology, image recognition, and other related majors were recruited in a targeted manner and subjected to the Ishihara Color Blindness Test, SAS test, and SDS test. A total of 244 undergraduates and master’s students were recruited at Sichuan University. After the selection, 25 participants had low professional relevance, 1 participant failed The Color Blindness Test, 9 participants exceeded the Chinese normative standard for SAS, and 3 participants exceeded the Chinese normative standard for SDS (3 of them exceeded both SAS and SDS normal level)^3^. After removing 35 participants, 209 subjects (101 males and 108 females) were identified as raters with an average age of 21.67 years. Thereafter, the raters were required to register online in the PES, sign an informed consent form, and receive a certain amount of payment upon completion of the experiment.

#### 3.1.3. Procedures

The 209 raters were randomly and equally divided into six groups, A_1_, A_2_, B_1_, B_2_, C_1_, and C_2_. Sixty nine sets of pictures were randomly selected as common pictures, and the remaining 1,200 sets of images were divided into six blocks. Since each group of raters was required to complete 269 sets of picture ratings, the assessments were conducted in five times, taking about 65 min for each evaluation.

Aiming at minimizing the influence of external environmental factors on the rating results, the experiment was conducted in a standard laboratory environment. During the experiment, the computer screen resolutions were uniformly adjusted to 1600*1024, while the curtains were closed and the lights turned on, and two experimenters maintained order in the site and answered any questions that arose in a timely manner.

Raters logged into the PES before the experiment started and autonomously watched the operation video (they could choose to skip the session in the next trial). Once the experiment started, raters were required to sequentially carry out affective self-reporting, pre-test practice, and judgment of the pictures, with the same procedure as Experiment 1.

### 3.2. Statistics

Similar to Experiment 1, we first calculated the internal consistency of the rater’s assessments using the Cronbach’s alpha coefficient. Meanwhile, considering the specificity of the data material, we referred to Loewenthal’s (2001) related study which used the mean correlation coefficient between individual ratings and overall ratings as the reliability test result. According to Cook et al. (2018), if the reliability result is greater than 0.5, we can conclude that the rater’s assessments are reliable.

In terms of image quality improvement, the images were first further screened based on the 60% identify ratio criterion. Furthermore, we compared the retained images’ labels in Experiments 1 and 2; after removing the images with inconsistent labels, we eventually built the image library.

### 3.3. Results

#### 3.2.1. Reliability measures

To verify the reliability of the rating results of raters, the affective ratings of 69 sets (a total of 400 pictures) of common pictures were analyzed separately, and after removing 10 images with missing data due to system problems, the internal consistency scores of the remaining pictures were calculated, and it was found that the Cronbach’s alpha of valence and arousal were 0.974 and 0.977, respectively.

Furthermore, referring to Loewenthal’s (2001) related study, we calculated the reliability again. The data showed that the reliability for valence and arousal were 0.795 and 0.61, respectively, which were greater than the discriminant criterion of 0.5 (Cook et al., 2018); thus, it can be assumed that the scoring results were somewhat reliable.

#### 3.2.2. Identity ratio of ratings

In accordance with the method adopted in Experiment 1, the affective ratings (9 points) were converted into affective labels (valence, arousal). After calculating the identity ratio of the valence ratings and arousal ratings for each image separately, a total of 4,149 pictures reached 60% criteria at the same time, including 497 images of people, 693 images of animals, 682 images of plants, 741 images of objects, and 1,536 images of scenes.

#### 3.2.3. Generate image library

Comparing the results of Experiment 1 and Experiment 2, the images with consistent affective labels were defined as valid pictures (such as positive-positive, low-low). The results showed that there were 3,910 images with consistent valence labels and 3,579 images with consistent arousal labels. After comprehensive consideration of the valence and arousal labels, a total of 3,386 valid pictures were obtained, which can be seen in Table 4.

**TABLE 4:** Descriptive statistics of the material library.

Category	Numbers	Valence			Arousal		
		Min.	Max.	Mean	Min.	Max.	Mean
People	404	1.00	8.97	5.04	1.21	8.94	5.80
Animals	585	2.27	8.09	5.72	2.32	8.71	5.20
Plants	529	2.88	8.02	5.94	2.23	7.42	4.95
Objects	609	1.92	8.21	5.33	1.13	9.00	4.63
Scenes	1,259	2.01	7.90	5.14	1.01	7.30	4.43

**FIGURE 3:**
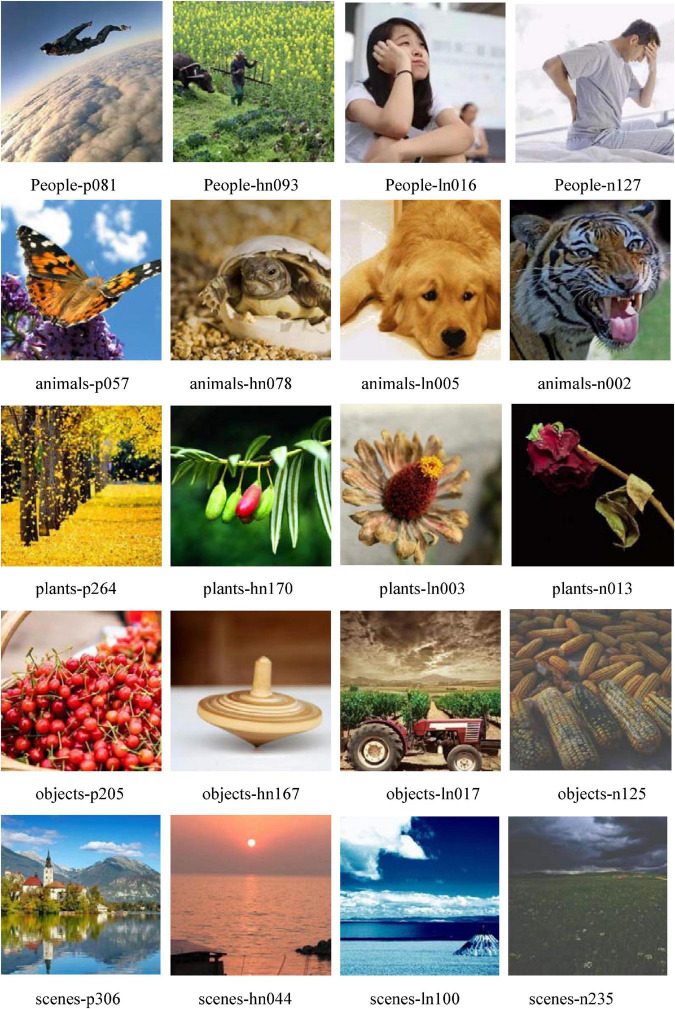
Example of the material library. In HDRPS, images are named according to content category, valence label, and number. For example, people-n081 represents the 81st positive picture of the people category. The rating for valence (V), arousal (A) are as follow: people-p081, V = 6.65, A = 7.30; people-hn093, V = 5.84, A = 4.96; people-ln016, V = 4.57, A = 3.94; people-n127, V = 2.67, A = 4.17; animals-p057, V = 6.11, A = 6.14; animals-hn078, V = 5.76, A = 4.98; animals-ln005, V = 4.46, A = 4.03; animals-n002, V = 3.38, A = 7.97; plants-p264, V = 6.70, A = 6.21; plants-hn170, V = 5.89, A = 5.03; plants-ln003, V = 4.05, A = 3.98; plants-n013, V = 3.47, A = 3.73; objects-p205, V = 6.74, A = 5.08; objects-hn167, V = 5.80, A = 4.98; objects-ln017, V = 4.45, A = 5.23; objects-n125, V = 2.78, A = 4.88; scenes-p306, V = 6.92, A = 6.16; scenes-hn044, V = 5.93, A = 4.83; scenes-ln100, V = 4.69, A = 4.02; scenes-n235, V = 2.98, A = 4.11.

With Experiment 1 and Experiment 2, a material library containing 3,386 pictures (the details can be seen in Figure 3) was established in this research. Experiment 3 would develop a picture-based scale that can be used for affective recognition based on the material library, and has initially validated the validity of the scale.

## 4. Experiment 3: Pre-test of the HDRPS

### 4.1. Methods

#### 4.1.1. Materials

Each picture of the material library had two dimensions, valence and arousal; the combination of pictures according to the type of valence and arousal, allowed the development of a scale with nine pictures presented in a row. Considering that showing too many pictures would make the selection more difficult and prolong the testing time, whereas the research on emotions in organizational settings mainly concentrated on positive and negative moods (Fisher and Ashkanasy, 2002), a picture-based scale was developed based on valence ratings. In the meantime, a validity verification system for picture-based scales (PSVS) was developed to facilitate the implementation of Experiment 3.

In the process of testing the picture-based scale, the PSVS randomly presented one type of pictures, and automatically selected 1 positive picture, 1 neutral picture, and 1 negative picture to generate the test scale; then, the participants were required to choose one from the three pictures that best matched their current state of affect/mood. Considering the possible problem of personal preference during the selection of pictures, five rounds of testing were repeated, with five categories of pictures presented randomly.

#### 4.1.2. Participants

In order to control factors such as age and cultural background on the test results of the picture-based scale as much as possible, 261 volunteers who did not engage in Experiments 1 and 2 were recruited to participate in the pre-test In line with the first two experiments, the color blindness test, SAS test, and SDS test were conducted sequentially, and a total of 229 volunteers passed the qualification screening. Participants who passed the selection process were required to register online by logging into the PSVS, signing an informed consent form, and receiving RMB 100 compensation upon completion of the experiment.

Watson et al. (1988) discovered that positive affective (PA) states showed a strong time-of-day effect: PA scores tended to rise throughout the morning, remain steady during the rest of the day, and then decline again during the evening. Accordingly, the participants were asked to take part in the pre-test at the same time every day for five consecutive days (from Monday to Friday) in order to overcome the time effect on the test results. For management purposes, we made an experimental schedule before the experiment began, fixing the daily experimental time into six time periods, and participants were allowed to choose the time in which they participated in the experiment according to their personal schedule, thus forming 6 test groups (a, b, c, d, e, f). Statistically, 209 participants (73 males and 136 females) joined the experiment throughout with an average of 20.58 years.

#### 4.1.3. Procedures

This experiment was conducted online in order to control the influence of the external environment on the participants’ affect and to maximize closeness to the real study and working scenarios. The day before the start of the experiment, we organized volunteers with the help of a Tencent Meeting and explained the experiment procedure and governance rules in detail.

At the beginning of the formal experiment, volunteers first registered for the Tencent meeting, opened the webcam for online check-in, and ensured that the webcam was on throughout the experiment. The experimenter used the video to understand the volunteers’ working and learning environment and ensure that the influences of external factors on the test results were within the controllable range. After signing in, participants logged into the PSVS, watched the demonstration video (the session could be skipped), and then entered the formal experimental session.

The formal experiment was composed of three panels. One is the calibration scale test; for validating the picture-based scale, the PANAS scale and SAM scale (valence) were taken as valid standards. The other is the practice; a total of five sets of practice pictures were prepared, and three sets were randomly selected by the system for the exercise (the next time this session can be skipped). The last one is the picture-based scaleHDRPS test, in which the system randomly presented five sets of pictures (1 set/category), and participants needed to select one picture from each of the three pictures that best matched their current emotions/mood, which took about 5 min for a single trial.

A total of 1,045 tests on the HDRPS were conducted in this experiment, and after removing missing data items, duplicate record items, and apparently abnormal items, 845 valid test results remained.

### 4.2. Statistics

#### 4.2.1. Reliability measures

Since the HDRPS was a single-item scale, the Cronbach’s alpha coefficient could not be calculated during the scale validation process, and the average correlation coefficient between each rating and the overall rating was used as the reliability test result by referring to Loewenthal (2001). As with Experiment 2, the results were considered to have internal consistency and pass the reliability measure if the calculated result was greater than 0.5.

#### 4.2.2. Validity measures

Validity measures refer to the degree to which an instrument measures what it intends to measure (Cook et al., 2018), typically using Pearson correlation proofs, which is the predominant form of validation for picture-based scales. Lynn (1986) pointed out that three types of validity are commonly used today: content, criterion-related, and construct. Because Experiments 1 and 2 had already ensured the content validity, this part mainly examined the validity of the criterion validity and construct validity.

Concurrent validity represents the degree to which the results of a test correspond to those of existing (previously validated) test(s) of the same construct at the same point in time (Cook et al., 2018), and usually the larger the value of concurrent validity, the better the newly developed scale is. Therefore, we used the concurrent validity to measure the criterion validity; and referring to the criteria proposed by Murphy and Davidshofer (2015), a result is considered significant if it is greater than 0.45.

Discriminant validity represents the extent to which measurements that are not expected to be related by theory are in fact unrelated or distinct (Cook et al., 2018), which is the opposite of concurrent validity, and the smaller the correlation coefficient, the better. Referring to the discriminant criterion proposed by Cook et al. (2018), a correlation coefficient of less than 0.45 is considered a passing validity.

### 4.3. Results

#### 4.3.1. Reliability measures

The reliability test result was calculated to be 0.70 meaning that the HDRPS passed the reliability test.

#### 4.3.2. Validity measures

First, the correlation between the SAM scale and the HDRPS was calculated, and the result showed a correlation coefficient of 0.633, which passed the concurrent validity test.

Given the fact that the PANAS scale measured the affective status of the most recent week, which belonged to a different construct from the HDRPS, the correlation coefficient was calculated as the discriminant validity. The result showed that the correlation coefficient was 0.394, which passed the discriminant validity test.

Following the Experiment 3, 30 subjects were invited to participate in semi-structured interviews to enhance the usefulness of the HDRPS, and eventually, 27 subjects completed online interviews with an average interview time of 15 min. To address the problem of difficult selection caused by relatively few neutral pictures, which was raised by 59.3% of the respondents, the presentation of the picture-based scale was adjusted, with neutral pictures further divided into two types: low neutral and high neutral (4 < valence ≤ 5 for low neutral, and 5 < valence ≤ 6 for high neutral).

## 5. Study 4: Formal test of the HDRPS

### 5.1. Methods

#### 5.1.1. Materials

Over the course of the experiment, the system randomly selected 1 category of pictures, and automatically extracted a set of pictures, which contained 1 positive picture, 1 high neutral picture, 1 low neutral picture, and 1 negative picture, to generate test item. The participants were required to select 1 picture that best matched their current state of affect/mood. As with Experiment 3, 5 rounds of test were set for each trial, and the 5 categories of pictures were presented randomly (specific picture examples are as follows).

People-p081 People-hn093 People-ln016 People-n127

animals-p057 animals-hn078 animals-ln005 animals-n002

plants-p264 plants-hn170 plants-ln003 plants-n013

objects-p205 objects-hn167 objects-ln017 objects-n125

scenes-p306 scenes-hn044 scenes-ln100 scenes-n235

#### 5.1.2. Participants

Working employees were recruited to participate in this experiment in order to test the validity of the HDRPS in real work settings, in which subjects were required to have normal color vision, normal or corrected visual acuity, normal mental functioning and to be capable of using the phone. Accounting for age differences in affect (e.g., Grühn and Scheibe, 2008), subjects were recruited in age groups and administered in subgroups.

This experiment recruited an aggregate of 636 participants. As the real work setting may have employees with mental abnormalities (e.g., SAS or SDS score over Chinese norm), the qualification screening only excluded employees with abnormal color vision. Statistics showed that altogether 522 participants passed the qualification screening and completed system registration. For gender, there were 154 males and 368 females. For age, 35.6% were aged 18–30 years, 27.6% aged 31–40 years, 23.9% aged 41–50 years, and 12.8% aged 51–60 years. For marital status, 33.7% were unmarried, 61.5% married and 4.8% divorced. For education, 28.7% were college graduates or below, 42.1% had undergraduate degrees, and 29.1% had master’s degrees or above. At the management level, government and institution employees accounted for 28.2%, enterprise employees accounted for 32.4%, service industry employees accounted for 6.5%, and workers, freelancers, and others accounted for 32.9%. Before starting the experiment, subjects signed an informed consent form online and were paid RMB 35 upon completion of the experiment.

#### 5.1.3. Procedures

The HDRPS is an affective measure tool that can be used repeatedly. To verify its continuous measurement efficacy, this experiment was conducted over a 7-day consecutive period (from Monday to Sunday). Because the experiment might interfere with the subjects’ normal working life, they were asked to log in to the PSVS on mobile before the end of the morning (11:30–12:30) and afternoon (17:30–18:30) for online testing following their own work schedules.

Due to the limited time and energy of participants, no further pre-experiment training was arranged. To ensure that participants were familiar with the experimental procedure, the testing function of PSVS was opened 2 days before the start of the experiment; thus, all participants could utilize their free time to log into the PSVS for any operation. Simultaneously, to verify the relation between pictures and emotions, we set up the calibration scale test before and after the HDRPS, and assessed whether the pictures could measure affect by comparing the difference between the first and second calibration scales.

The formal experiment consisted of three sections. For the first test of the calibration scales, participants needed to fill in the PANAS scale and SAM scale (valence) in turn after logging into the system. Then, for the HDRPS test, the system randomly presented 5 sets of images (1 set/category), and participants were required to select 1 picture from each of the 4 pictures that best matched their current state of affect/mood. Finally, the participants were asked to report their own valence level, again using the SAM scale, which took about 3 min for a single trial.

### 5.2. Statistics

#### 5.2.1. Validation of picture-emotion reflection mechanism

Pictures can be used as a standard material to induce emotions, and to verify whether pictures can be used as a tool to measure affect, the SAM scale was tested before and after the HDRPS test in Experiment 4.

We used two methods to verify the picture-emotion reflection mechanism. One is a paired-samples *T*-test of SAM results before and after the HDRPS test; we believed that if HDRPS stimulated subjects’ emotions, there should be a significant difference in the test results. On the contrary, if HDRPS can reflect subjects’ emotions, the results are not significant.

In addition, we referred to the relevant studies on affective arousal by Martin (1990), which defined the criterion for successful affect elicitation as a change in mood from pre-induction to post-induction of at least 10 percentage points. In our opinion, if the change in SAM before and after the HDRPS test is less than 10%, the picture-emotion reflection mechanism can be verified again.

#### 5.2.2. Reliability and validity of HDRPS

As in Experiments 2 and 3, the reliability test was conducted by using the same method as Loewenthal (2001)’s. At the same time, in addition to the construct validity by using discriminant validity, the criterion validity was conducted by using concurrent validity.

#### 5.2.3. Accuracy and stability of HDRPS

The HDRPS is a highly dynamic instrument for repeatedly measuring subjects’ affect changes, which can replace lengthy vertical scales and picture-based scales with fixed content to assess one’s affective rapidly. Therefore, to verify the validity of the HDRPS, the accuracy rate was calculated based on the 1st SAM scale test results.

Besides, considering that the HDRPS can be reusable to measure employees’ affect, we further investigated the stability of continued use by referring to a related study by Burr et al. (2021), which used the root Mean Successive Square Difference (rMSSD) to measure affective instability.

### 5.3. Results

#### 5.3.1. Validation of picture-emotion reflection mechanism

First, the paired-samples *t*-test of SAM results was performed (see Table 5 for details), and it was found that there was no significant difference (Sig > 0.05) in the participants’ valence rating before and after the HDRPS test.

**TABLE 5:** Paired-samples *t*-test of SAM.

	Mean	SD	SEM	95% CI of the difference		Sig. (2-tailed)
				Lower	Upper	
1st SAM and 2nd SAM	0.013	0.557	0.009	0.004	0.030	0.136

SD is the standard deviation, SEM is the pairing difference Standard Error Mean, CI is the confidence interval.

Based on this, with reference to Martin (1990)’s study, we calculated the magnitude of change and noticed that 79.05% of the participants had a change in affect of less than 10%, indicating that there was indeed a reflection mechanism between pictures and affect, and individual emotions can be measured with the help of pictures.

#### 5.3.2. Reliability and validity of HDRPS

In this experiment, a total of 4,339 tests of the HDRPS were conducted, and after deleting missing data items, duplicate record items, and apparently abnormal items, 3,778 valid test results remained. Referring to Loewenthal (2001)’s study, the mean correlation coefficient between each rating and the overall rating was calculated; and the result showed that the reliability test result was 0.54, which was greater than the judgment criterion of 0.5, therefore, we considered that the HDRPS scale passed the reliability test.

To verify the validity of the HDRPS, the correlation between the HDRPS and the calibration scales were calculated separately, and the results showed that the correlation coefficient between the HDRPS and the SAM scale was 0.45 and the correlation coefficient with the PANAS scale was 0.21, which passed the validity test.

#### 5.3.3. Accuracy and stability of HDRPS

According to the rating-affective label conversion method used in Experiment 1 and Experiment 2, we converted the results of the 1st SAM scale and the HDRPS into affective labels, and calculated the consistency of the test results. The data showed that the consistency ratio between the 1st SAM scale and the HDRPS was 89.73%, illustrating that the picture-based scale can relatively accurately measure employees’ affect.

By calculating the rMSSD of the PANAS scale, SAM scale, and the HDRPS separately and correlating them (see Table 6), we noticed that age, seniority, and marital status were negatively correlated with the affective instability of each scale (approved by Grühn and Scheibe, 2008), while SDS and SAS were positively correlated with affective instability. In this regard, we can conclude that the HDRPS was basically consistent with the test results of the calibration scales, which proved that the picture-based scale has a certain degree of scientificity and validity in continuously measuring employees’ affect.

**TABLE 6:** Analysis of affective instability.

Scale	rMSSD_PANAS_	rMSSD_1stSAM_	rMSSD_HDRPS_	rMSSD_1stSAM_
Gender	0.015	-0.042	0.039	-0.003
Age	-0.258**	-0.196**	-0.240**	-0.160**
Management level	-0.061	-0.104*	-0.106*	-0.073
Seniority	-0.126*	-0.147**	-0.130**	-0.117*
Marital status	-0.188**	-0.154**	-0.122*	-0.149**
Education	0.205**	0.055	0.107*	0.037
SDS	0.048	0.107*	0.106*	0.099*
SAS	0.095	0.113*	0.117*	0.116*

rMSSD is the root Mean Successive Square Difference. Star means that there is a significant relationship between factors.

*Means significant at 0.05 level and **means significant at 0.01 level.

## 6. Discussion

### 6.1. Research content

Affective measure is basic and essential for the development of affective science research. The commonly used methods of affective access are physiological indicator measures, external behavioral measures, and self-report methods. Currently, researches on physiological and behavioral-based affective recognition are mainly limited to laboratory settings, whereas emotional access in real work scenarios is primarily performed by verbal scales. Although a wealth of information can be obtained from verbal scales, there are some obvious problems. In the first place, the mature scales are all from Western studies, which makes it difficult to find exact equivalents of English words in the translation process (Watson et al., 1988). In the second place, the scales are generally applicable to Western cultures, and overly direct measures may lead subjects to respond so as to meet the requirements of the experiment. In the third place, affect is a transient state of the organism, thus the reliability of a single test is not high, while multiple tests are susceptible to learning effects. Last but not least, the semantic scales are comparatively demanding for the participants and are not applicable to less educated groups.

This article is devoted to developing a highly dynamic and reusable measurement tool for assessing individuals’ true affect. To guarantee the scientific and rational construction of the image materials, we designed a rigorous experimental process. At first, we gathered high-quality color pictures on the Internet, and the initial quantity of pictures reached 22,054. After standardized processing and usability evaluation, there were 8,933 pictures left. Next, 14 Ph.D students in related fields were invited to participate in Experiment 1 to judge the affective labels of pictures; after screening out the pictures with less than 60% identification ratio, 5,046 pictures were left. Then, 209 professional assessors were recruited to rate the pictures in Experiment 2, and 3,910 images with consistent valence labels and 3,759 images with consistent arousal labels were left after removing the pictures with less than 60% criteria. Based on this, we calculated the mean ratings of valence and arousal of each image as the picture affective rating, compared the results of Experiment 1 and Experiment 2, retained the pictures with consistent affective labels, and finally, built an material library with a capacity of 3,386 pictures.

Valence (pleasure) is one of the most basic evaluation features (Dan-Glauser and Scherer, 2011). Considering that research on affect in organizational settings has focused on positive affect and negative affect (Fisher and Ashkanasy, 2002), the present work developed a picture-based scale based on the valence label. For validating the picture-based scales, Experiment 3 and Experiment 4 were conducted successively. In Experiment 3, 209 volunteers joined the pre-test of HDRPS, and a total of 845 trials were effectively completed. Considering that the volunteers who participated in Experiment 3 were all college students with similar academic backgrounds and ages, the validity of the HDRPS in the organizational context could not be effectively verified. Hence, we recruited 522 working staff (aged 18–60) to participate in Experiment 4, and after 7 consecutive days of twice daily continuous measurement, we completed a total of 3,778 valid trials. Analysis of the results revealed that the HDRPS has good reliability which can be used to repeatedly measure subjects’ affect.

### 6.2. Innovation and limitations

The fourth industrial revolution accelerates the pace of people’s work, and the sudden increase of work stress has become a fertile ground for the breeding of negative affect, which seriously influences the work and life of employees. This article is dedicated to developing a picture-based scale that is highly dynamic and can reusable access subjects’ affect. Compared with previous affective measure methods, the new instrument has three features. Using pictures as a measurement instrument provides a way of perceiving affect without relying on semantic scales, and this method is relatively novel and concealed, which can reduce the subjects’ defensiveness. Besides, HDRPS relies on a large capacity material library, which can be randomly combined to generate test items on different topics, and enable highly dynamic measurement of emotions while controlling external factors such as preferences. In addition, HDRPS relies on cell phones, computers and other media for measurement, which is easy to use, simple and quick to operate (a single test time is less than 5 s). Therefore this approach is less likely to place an additional burden on the subject and can be applied to the continuous measurement of daily emotions.

The current study, like other studies, has some shortcomings. Foremost, the picture material only contained two dimensions of valence and arousal, thus dominance was not included. Empirical studies have shown the weakness of dominance in explaining overall variance in affect (e.g., Ferré et al., 2012), and that two-dimensional affective models are superior to models containing more dimensions. Some researchers have eliminated the dominance dimension in order to shorten the experimental time and reduce subject fatigue (such as Li et al., 2012). However, future studies still need to consider the role of dominance in the material database.

Another limitation is that, during the development of the HDRPS, to suit the reality of management and reduce the difficulty of selecting, we extracted the pictures to form the test items based on valence labels without considering the arousal dimension. Therefore, future research should consider how to use the same thematic image to reflect two different affective dimensions and to measure subjects’ affective more comprehensively.

Besides, HDRPS is completely built in China, and we have yet to investigate how cultural differences may affect the score of pictures, or even whether this method would be appropriate to apply cross-culturally. Future studies using HDRPS should be aware of the possible effects of cultural differences. Since we have provided scores for valence and arousal of all images, we encourage future studies to perform small-scale validation before using this tool.

Then again, the number of measurements can be decided autonomously during the access of the HDRPS. In this study, we repeated the picture selection 5 times during the validation of the scale to prevent the influence of personal preference on the results, which prolonged the testing time, hence a single trial can be conducted in the later study to compare the difference in accuracy between a single measurement and multiple measurements.

A further limitation is that the capacity of the material library is 3,386, among which 11.93% are people, 17.28% are animals, 15.63% are plants, 17.99% are objects and 37.18% are scenes, showing an uneven distribution. Future research should continue to explore picture-based scales with a more balanced structure distribution.

## Data availability statement

The original contributions presented in this study are included in the article/supplementary material, further inquiries can be directed to the corresponding author.

## Ethics statement

Written informed consent was not obtained from the individual(s), nor the minor(s)’ legal guardian/next of kin, for the publication of any potentially identifiable images or data included in this article.

## Author contributions

PL was the person in charge of the whole project research. YW was the research designer and implementer. J’nH was responsible for the implementation of experiments and data analysis. L’bQ was responsible for guiding the technical development of the system. KZ was responsible for the design and development of the system. All authors contributed to the article and approved the submitted version.

## Footnotes

1
http://syy.imagesoft.cc:8083/#/evaluatePhase

2
http://syy.imagesoft.cc:8083/#/testPhase

3 The retention rate of images in the rating session was 56.49%, and since only one picture was left in some sets, it was not significant to group statistics, so the data were not counted in groups thereafter.
